# Bisected full-thickness skin graft for reconstruction after Mohs micrographic surgery

**DOI:** 10.1016/j.jdin.2024.08.008

**Published:** 2024-09-07

**Authors:** Hoon Choi, Jun Ho Kwak, In Ho Bae, Chan-Ho Na, Bong Seok Shin, Min Sung Kim

**Affiliations:** Department of Dermatology, Chosun University College of Medicine, Gwangju, Republic of Korea

**Keywords:** basal cell carcinoma, cosmetic, Mohs micrographic surgery, reconstruction, skin cancer, skin graft, squamous cell carcinoma

*To the Editor:* There are several options for reconstructing skin defects after excision of skin cancer. Full-thickness skin grafts (FTSG) are used when other methods are not feasible.^1^ FTSG offers versatility in size and shape for improved cosmetic outcomes but is limited by donor site size.^2^ We introduced bisected FTSG (bFTSG) for larger skin defects.

We analyzed 23 patients who underwent Mohs micrographic surgery with bFTSG at Chosun University Hospital from April 2014 to March 2023. A tailored template for the defect was halved along the long axis. Two divided templates were placed in a row on the donor site. After harvesting, the 2 grafts were sutured to fit the defect with horizontal mattress sutures, and then tie-over bolster dressing composing polyurethane with silicone membrane (Mepilex border) was applied on the recipient (Fig 1). One week after surgery, the bolster dressing and stitches were removed.

**Fig 1:**
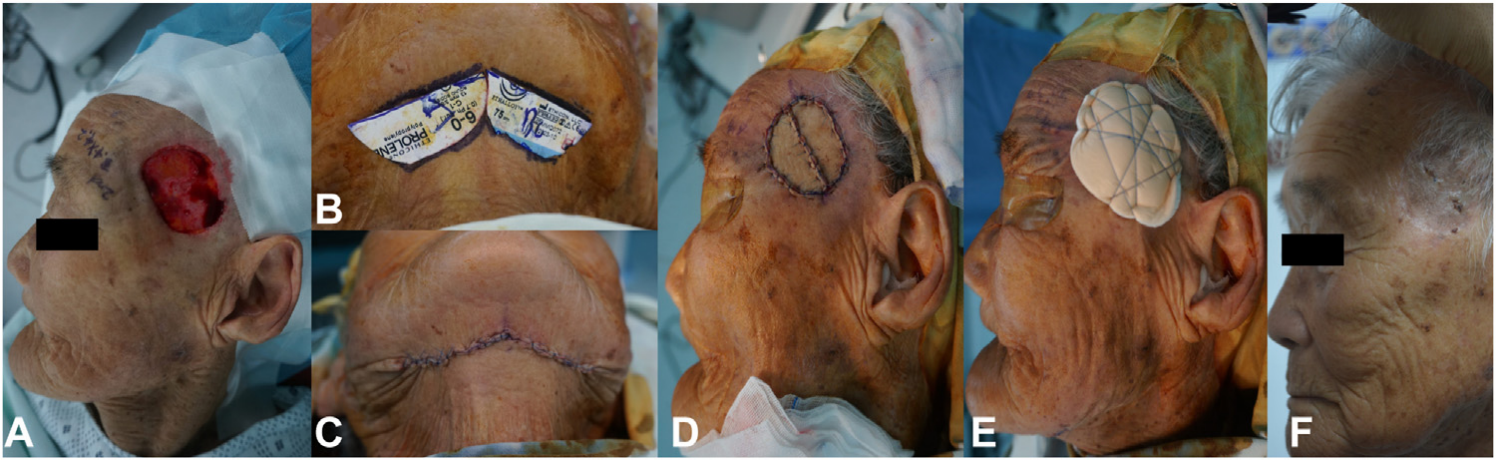
Bisected Full-thickness skin grafts. **A,** Skin defect status after Mohs micrographic surgery. **B,** A template of the same size as the defect is divided in half and designed on the donor area, the submandibular area. **C,** A full-thickness incision of donor area was performed, and the donor site was sutured. **D,** Two skin grafts were placed in the defect according to outline and were sutured. **E,** Tie-over bolster dressing was applied on the recipient. **F,** Postoperative site 1 month after surgery.

Factors such as patient demographics, tumor, and surgical details were analyzed (Table I). The anticipated reduction in scar length at the donor site was calculated by subtracting the long axis of the actual scar diameter from the expected scar diameter in FTSG. The 3:1 elliptical design used in FTSG results in a scar approximately 3 times the length of the defect, which can lead to large donor defects. Previous studies have sought ways to minimize the size of the recipient site for larger surgical defects.^2^, ^3^, ^4^ Based on the findings, we have introduced a modified approach known as bFTSG.^2^, ^3^, ^4^ When performing bFTSG, the scar length at the donor site was expected to be reduced by 3.5 cm compared with the 3:1 elliptical design. The donor site scar in bFTSG is approximately twice the length of the long axis of the defect because the donor defect is designed by arranging the templates, which are divided in half along the long axis, in a row. Additionally, bFTSG resulted in minimal scarring with Vancouver Scar Scale score of 4.2 in our study. However, additional sutures are required to connect 2 divided grafts to ensure proper alignment and fixation.

**Table I:** Patient demographics and tumor and surgical characteristics

Variables	Overall, *N* (%)
No. of patients	23 (100)
Age, y (mean [SD])	82.9 (8.6)
Sex	
Male	8 (34.8)
Female	15 (65.2)
Comorbidities	
Hypertension	13 (56.5)
Diabetes mellitus	2 (8.7)
Hyperlipidemia	2 (8.7)
Dementia	2 (8.7)
Parkinson disease	2 (8.7)
Major depressive disorder	2 (8.7)
Others∗	1 (4.3)
Pathologic diagnosis	
Squamous cell carcinoma	13 (56.5)
Basal cell carcinoma	7 (30.4)
Pleomorphic sarcoma	2 (8.7)
Melanoma	1 (4.3)
Tumor location	
Head and neck	19 (82.6)
Forehead	3 (13.0)
Temple	6 (26.1)
Cheek	5 (21.7)
Periauricular	2 (8.7)
Periorbital	3 (13.0)
Upper extremity	1 (4.3)
Lower extremity	3 (13.0)
Size of tumor, long axis, cm (median [SD])	2.5 (1.0)
MMS stages, number, (mean [SD])	1.9 (1.0)
Size of the skin defect, long axis, cm (mean [SD])	4.1 (1.2)
Donor site	
Submandibular	16 (66.7)
Gluteal line	1 (4.3)
Postauricular area	2 (8.3)
Arm	2 (8.3)
Ankle	2 (8.3)
Anticipated reduction in scar length at donor site, long axis, cm (mean [SD])	3.5 (3.5)
Complications	
None	17 (73.9)
Inflammation	5 (21.7)
Recurrence	1 (4.3)
Vancouver scar scale at the time of 1 month after the surgery	
Total score	4.2 ± 2.0
Vascularity	0.9 ± 0.9
Pigmentation	1.2 ± 1.0
Pliability	0.9 ± 0.7
Height of depression	1.3 ± 0.6

*MMS*, Mohs micrographic surgery.

∗ Others include benign prostatic hyperplasia.

The nasolabial fold, eyelid, preauricular, postauricular, and supraclavicular areas are common donor sites for facial defect.^1^^,^^5^ However, these sites are not suitable for large defects. We selected the submandibular area as the donor site for large facial defects. Submandibular harvesting yields a larger amount of skin because of age-related skin loosening. It also conceals potential scars that could be anatomically hidden by jaw. Additionally, the subjective improvement in chin contour was noted with suture tightening of the donor site.

The average size of the long axis of the skin defect was approximately 4.1 cm, indicating bFTSGs effectiveness for larger defects. Although 5 patients experiencing an inflammatory reaction and 1 patient having a recurrence, the procedure was well tolerated with minimal side effects. Despite the small sample size of the study, bFTSG offers significant advantages in skin defect reconstruction, including reduced donor site scar length, suitability for larger defects, and improved cosmetic outcomes. Therefore, bFTSG is a well-tolerated option for skin reconstruction.

## Conflicts of interest

None disclosed.
